# Dendrimer-conjugated isotretinoin for controlled transdermal drug delivery

**DOI:** 10.1186/s12951-023-02052-5

**Published:** 2023-08-21

**Authors:** Tianqi Zhao, Mingwei Zhou, Ronghui Wu, Huaxin Wang, Christos C. Zouboulis, Mingji Zhu, Myongsoo Lee

**Affiliations:** 1 Department of Dermatology, China-Japan Union Hospital of Jilin University, Changchun, 130031 Jilin China; 2 Department of Chemistry, Fudan University, Shanghai, 200438 China; 3 Departments of Dermatology, Venereology, Allergology and Immunology, Dessau Medical Center, Brandenburg Medical School Theodor Fontane and Faculty of Health Sciences Brandenburg, Dessau, Germany

**Keywords:** Dendrimer, Isotretinoin, Nanoconjugate, Transdermal drug delivery, Acne, Psoriasis

## Abstract

**Background:**

In the present study, we aimed to develop a novel isotretinoin delivery model for treating skin diseases, revealing its potential advantages in drug delivery and targeted therapy.

Using a self-assembly strategy, we grafted a dendrimer, based on a well-defined branched structure for nanomedical devices, with a well-defined nanoarchitecture, yielding spherical, highly homogeneous molecules with multiple surface functionalities. Accordingly, a self-assembled dendrimer-conjugated system was developed to achieve the transdermal delivery of isotretinoin (13cRA-D).

**Results:**

Herein, 13cRA-D showed remarkable controlled release, characterized by slow release in normal tissues but accelerated release in tissues with low pH, such as sites of inflammation. These release characteristics could abrogate the nonteratogenic side effects of isotretinoin and allow efficient skin permeation. Moreover, 13cRA-D exhibited high therapeutic efficacy in acne models. Based on in vitro and in vivo experimental results, 13cRA-D afforded better skin penetration than isotretinoin and allowed lesion targeting. Additionally, 13cRA-D induced minimal skin irritation.

**Conclusion:**

Our findings suggest that 13cRA-D is a safe and effective isotretinoin formulation for treating patients with skin disorders.

**Graphical Abstract:**

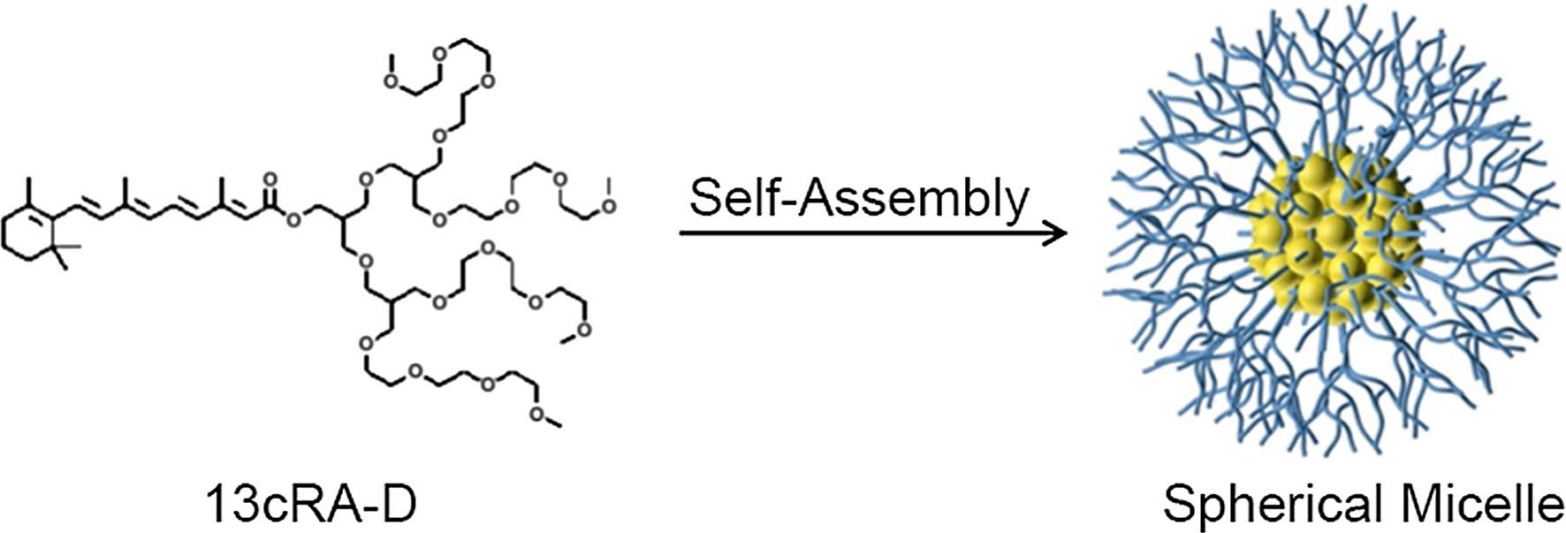

## Background

Isotretinoin (also known as 13-cis retinoic acid) is the first-choice treatment for severe acne [1–3] and is used off-label for treating other skin conditions [4]. However, oral isotretinoin administration has been associated with several skin-related adverse effects [2, 4], including depression, cheilitis, xerosis, and retinoid dermatitis [2, 5]. Furthermore, isotretinoin has a limited half-life owing to its poor stability and loss of activity during storage [6]. Topically applied isotretinoin has the advantage of negligible systemic absorption [7], which is important for safe treatment owing to the major side effect associated with systemically administered retinoids, namely teratogenicity [2, 5]; however, topical formulations are sensitive to oxygen when exposed to air, light and acids.

Recently, transdermal delivery approaches have substantially contributed to the field of medicine [8]. However, physiological skin barriers limit the use of small molecule and lipophilic drug candidates [9]. To broaden the application of transdermal delivery systems, novel approaches featuring microneedles and drug carriers, such as liposomes and nanoparticles, have been developed [10–13]. However, these approaches are restricted owing to drawbacks such as low bioavailability, frequent dosing or unstable delivery profiles [9, 14], patient discomfort due to reversible disruption of the stratum corneum by microneedles and complex procedures for achieving enhanced delivery across the stratum corneum [15–18]. To optimize dermatological therapeutic strategies, it is crucial to explore suitable pharmacokinetics and isotretinoin accumulation in the epidermis, potentially establishing a "depot-like" environment for controlling delivery [19].

Dendrimers are hyperbranched materials that belong to a new class of polymers with unique properties [20–22]. Dendrimer-based drug delivery systems have a wide range of biomedical applications owing to their tunable physicochemical properties [23]. Dendrimers with hydrophobic and hydrophilic exteriors, similar to lipid bilayer membranes, can encapsulate distinct types of hydrophobic cargo for cellular delivery [24].

In the present study, we synthesized dendrimer-conjugated isotretinoin (13cRA-D) and evaluated its ability to reduce side effects, enhance transdermal drug delivery and improve the therapeutic effect of isotretinoin.

## Material and methods

### Materials and reagents

Isotretinoin was obtained from APExBIO Technology (Houston, TX, USA) and applied to cells under dimmed yellow light. Advanced MEM, phosphate-buffered saline (PBS, 10 ×), antibiotic–antimycotic solution, fetal bovine serum (FBS), and l-glutamine solution (100 mM) were purchased from Sigma-Aldrich (St. Louis, MO, USA). The SZ95 human sebaceous gland cells [25] were cultured in Dulbecco’s Modified Eagle Medium (Gibco, USA), containing 10% FBS (Gibco, USA), 100 U/mL penicillin and streptomycin (Gibco, USA) in an incubator at 37 ℃ under 95% air and 5% CO_2_. Imiquimod cream was purchased from Sichuan Mingxin Pharmaceutical (Chengdu, China). The hydrophilic oligoether dendrimer was synthesized as reported previously [26–29].

### ^***1***^H-NMR measurement

An FT-NMR spectrometer was used to obtain ^1^H NMR spectra at 500 MHz. We used parts per million (ppm) to express the chemical shifts compared to the tetramethylsilane or residual solvent peaks (CDCl_3_:^1^H, 7.26). The indices s (singlet), d (doublet), and t (triplet) represent the multiplicity. Hz indicated the coupling constants.

### Transmission electron microscopy (TEM)

To examine the self-assembled constructions, one drop of each sample solution was placed on a 200 mesh carbon-coated copper grid with Formvar (Carbon Type B [15–25 nm]; Ted Pella, Inc.) and then evaporated under surrounding conditions. We then performed negative staining of samples by depositing a drop of uranyl acetate aqueous solution (0.2–0.4 wt %) on the grid surface of loaded samples. For positive staining, the sample-loaded grids were exposed to RuO_4_ vapor, generated by reacting a mixture of RuCl_3_⋅3 H_2_O (0.2 g) and 5% NaClO (10 mL) for 5 min. A JEOL JEM HR2100 instrument operating at 120 kV was used to observe the dried specimens.

### Analysis of dendrimer toxicity

Dendrimer toxicity was evaluated using Cell Counting Kit-8 (CCK-8). Prepared SZ95 sebocytes were treated with gradient concentrations of the dendrimer (0.025, 0.05, 0.1, 0.2 or 0.4 mg/mL) for 24 h. Next, CCK-8 reagent was added to the plates and incubated with cells for 1 – 2 h at 37 ℃ and under a humidified 5% CO_2_ atmosphere. Finally, absorbance was measured at 450 nm using a plate reader.

### Synthesis of 13cRA-D

A mixture of 300 mg isotretinoin (13cRA; 1 mmol), 290 mg dendrimer (0.3 mmol), 45 mg 4-dimethylaminopyridine (0.37 mmol), and 82 mg dicyclohexylcarbodiimide (0.4 mmol) was maintained under stirring in dry toluene (8 mL) at 0 ℃ for 1 h. The reaction was performed for 3 days. After completion of the reaction, the reaction mixture was quenched with water and extracted using ethyl acetate following thin-layer chromatography. The brine used to wash the organic layer was then dried over anhydrous MgSO_4_. After the filtrate was condensed under reduced pressure, the crude product was purified by silica gel flash column chromatography using methanol:ethyl acetate (1:50) as the eluent to yield a 26% (100 mg) yellow liquid.

### In vitro* release study*

We analyzed 13cRA-D release in vitro using a dialysis membrane (molecular weight cutoff = 1000 kDa). 13cRA-D was sealed in a dialysis bag and immersed in methanol (pH 5.0 or 7.0) at 37 ℃. Methanol was collected at 0.5, 1, 2, 4, 8, 12, 24 and 48 h. Then, methanol was measured using an ultraviolet–visible spectrophotometer to assess drug release. After the removal of each sample, 1 mL of fresh solvent was added to maintain a constant volume.

### Ethical approval

Animal experiments were performed according to the guidelines for the Care and Use of Experimental Animals of Jilin University and were approved by the Animal Experiment Ethics Committee of Jilin University.

### Skin permeation test

For transdermal testing, we employed the skin of Yorkshire pigs (1-month-old). We carefully excised the subcutaneous tissue to obtain an intact epidermis and then stored the skin at − 80 ℃ until use. The skin was cut into slices (approximately 30–30 mm^2^) and soaked in PBS at 37 ℃ for 1 h before use. Experiments were performed using a Franz diffusion cell at 37 ℃ and 100 rpm, avoiding light exposure. Then, 30% isopropanol and 70% PBS were added to receptor cells. The donor cells contained PBS, 0.25 mg/mL 13cRA, an equal weight of 13cRA-D comprising 0.25 mg/mL 13cRA or an equal weight of dendrimer. The prepared skin was placed between the receptor and donor cells. Samples were collected from the side arms at 2, 4, 6, 8, and 10 h to perform high-performance liquid chromatography (HPLC) measurements. All experiments were performed in triplicate.

### Skin retention test

After observing skin penetration, skin samples were removed from the Franz diffusion cell, cut into small pieces of (~ 1 mm × 1 mm) and homogenized in a 20 mL mixture of chloroform:methanol (1:2) to extract the retained drug, which was measured using HPLC.

### Drug distribution and retention

Following the skin penetration study, skin samples were cut into small pieces (~1 mm × 1 mm) and fixed in 4% paraformaldehyde overnight. Next, the samples were embedded in paraffin and sliced into approximately 4 μm tissue sections. Finally, mounted sections were observed under a fluorescence microscope, and images were analyzed using ImageJ software (National Institutes of Health, Bethesda, MD).

### Skin irritation test

The notum hair of 12 BALB/c mice (six males and six females, 20–30 g) was carefully removed, ensuring the epidermis remained intact. Mice were randomly divided into four groups treated with PBS, dendrimer, 13cRA or 13cRA-D, applied to the exposed skin; the applied substance was carefully removed with cotton after 6 h. Finally, the skin was scored (0–4) according to a published standard for evaluation [30]: 0, no erythema; 1 slight erythema; 2 moderate erythema; 3 moderate-to-severe erythema; 4, severe erythema.

### Treatment effect in acne model

To construct the Kligman acne model [31], we applied coal tar to the ear tubes of 12 of 15 New Zealand white rabbits (males, 2–2.5 kg) once daily for 14 days. On day 15, the rabbits were randomly divided into four groups, which were administered PBS, dendrimers, 13cRA or 13cRA-D respectively, once daily until day 28. Next, we evaluated the gross morphological and histological changes. Acne severity was evaluated using the above-scored standards. We performed hematoxylin and eosin (H&E) staining to assess the development of hair follicle horn plugs.

### Psoriasis model

We randomly divided 20 female BALB/c mice (20–30 g) into four groups and removed notum hair using a depilatory cream. Imiquimod cream (Sichuan Mingxin Pharmaceutical, Chengdu, China) was evenly applied daily to 15 of the 20 mice for seven days. We applied gentle pressure for 30 min until the cream was fully absorbed. Six hours after each application, PBS, 13cRA or 13cRA-D was applied for 30 s. Subsequently, we assessed the skin for gross morphological and histopathological changes. Skin inflammation severity was assessed using the Psoriasis Area Severity Index (PASI) [32]. Subsequently, we adjusted the mouse model based on evaluation using the clinical PASI score. The affected skin area was not considered in the overall score evaluation. Erythema and scaling were evaluated using a scale of 0 to 4 as follows: 0, none; 1, slight; 2, moderate; 3, marked; 4, very marked. Pathological changes in the psoriasis-like model were observed using H&E staining and Baker scoring, ranging from 0 to 10, under a light microscope [33].

### Statistical analysis

Data are presented as mean ± standard deviation (SD). Statistical analyses were performed using analysis of variance (ANOVA) or two-sample Student’s *t*-tests, followed by the least significant difference post hoc test. A *p-*value < 0.05 indicated a statistically significant difference between groups.

## Results

### Chemical synthesis of 13cRA-D

**Fig. 1:**
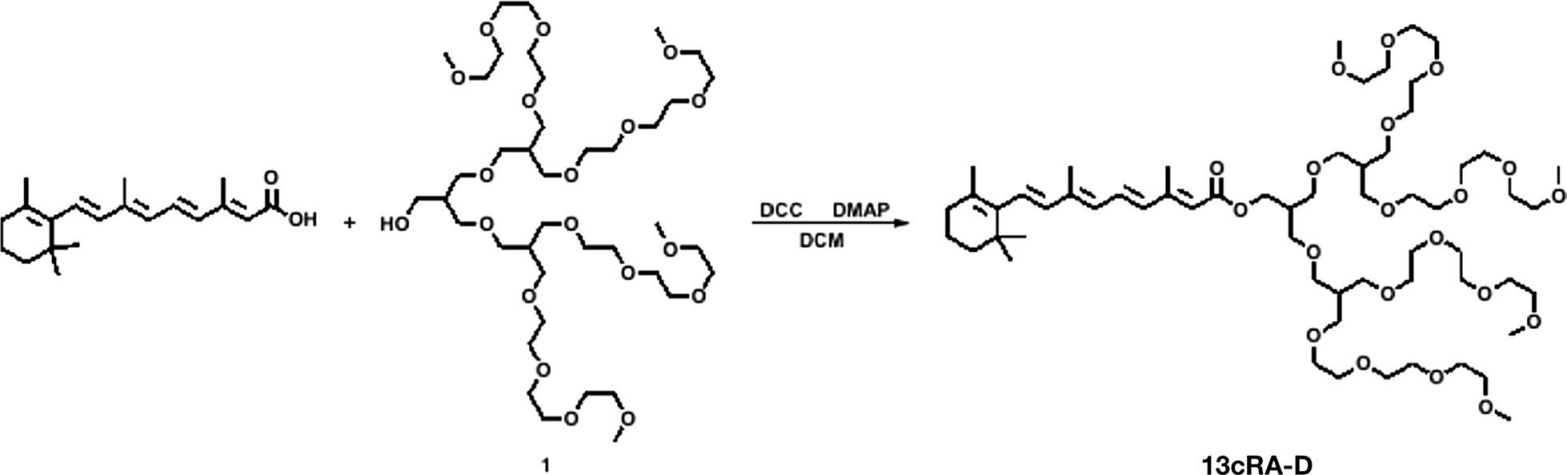
Synthesis of 13cRA-D

Figure 1 illustrates the synthesis of 13cRA-D by esterification of isotretinoin and a hydroxy-terminated dendrimer. The tetra-branched dendrimer was synthesized in a stepwise manner, initially by the etherification of 3-chloro-2-chloromethyl-1-propene with linear tri-ethylene glycol monomethyl ether, followed by hydroboration to obtain a di-branched chain. The resulting hydroxy-terminated di-branched chain was subjected to repeated reactions to obtain a tetra-branched dendrimer, esterified with 13cRA to yield 13cRA-D [26–29]. The obtained product was characterized by ^1^H-NMR and was in full agreement with the expected chemical structure. 13cRA-D is amphiphilic, given the presence of hydrophobic isotretinoin and a hydrophilic dendrimer, and is capable of self-assembling into nanostructures in an aqueous solution comprising a hydrophobic isotretinoin core surrounded by hydrophilic dendrimer segments. The ester linkage grafting of isotretinoin and the hydrophilic dendrimer can be cleaved by altering pH from neutral pH, thereby facilitating isotretinoin release after tissue delivery at low pH.

### ^1^H NMR & ^13^C NMR data of 13cRA-D

^1^H NMR (400 MHz, DMSO-*d*_6_) δ 7.66 (d, *J* = 15.2 Hz, 1H), 7.07 (dd, *J* = 15.3, 11.5 Hz, 1H), 6.34–6.16 (m, 3H), 5.69 (s, 1H), 4.07 (d, *J* = 5.9 Hz, 2H), 3.54–3.34 (m, 64H), 3.23 (s, 12H), 2.15 (m, 1H), 2.09–1.95 (m, 10H), 1.69 (s, 3H), 1.62–1.53 (m, 2H), 1.48–1.41 (m, 2H), 1.02 (s, 6H).

^13^C NMR (101 MHz, DMSO) δ 140.55, 137.60, 137.42, 134.47, 133.57, 133.11, 131.19, 130.11, 129.39, 128.90, 126.86, 116.45, 71.77, 70.45, 70.33, 70.28, 70.14, 70.09, 69.27, 69.02, 58.52, 40.65, 40.45, 40.24, 40.03, 39.82, 39.61, 39.40, 34.33, 33.11, 29.27, 21.98, 20.94, 19.19, 13.12.

### Characterization of 13cRA-D

**Fig. 2:**
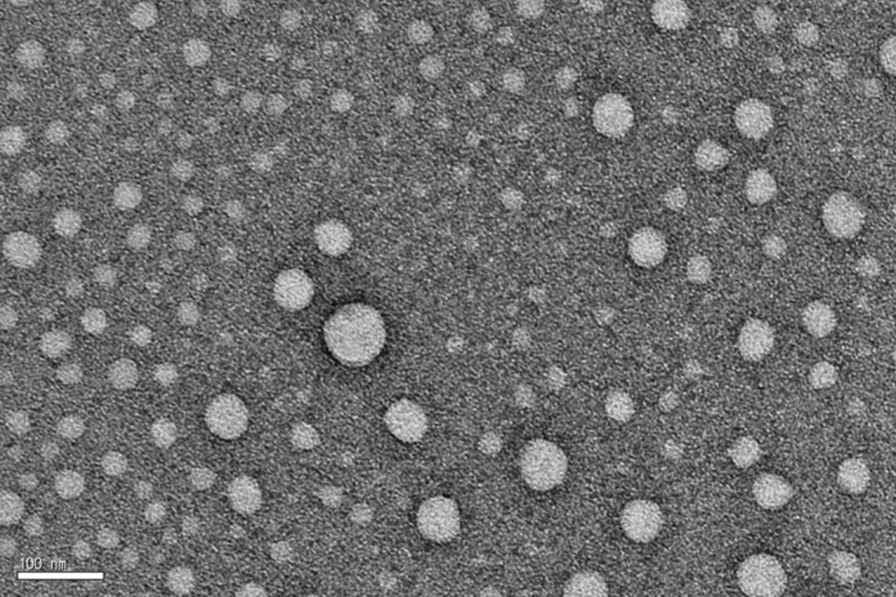
Negatively-stained transmission electron microscopy image of spherical nanostructures from 0.01 wt% aqueous solution of 13cRA-D. Scale bar: 100 nm

The formation of self-assembled isotretinoin amphiphile nanostructures was examined using negatively-stained TEM images (Fig. 2). The images show spherical nanostructures with an average diameter of ~ 25 nm.

### Dendrimer toxicity

**Fig. 3:**
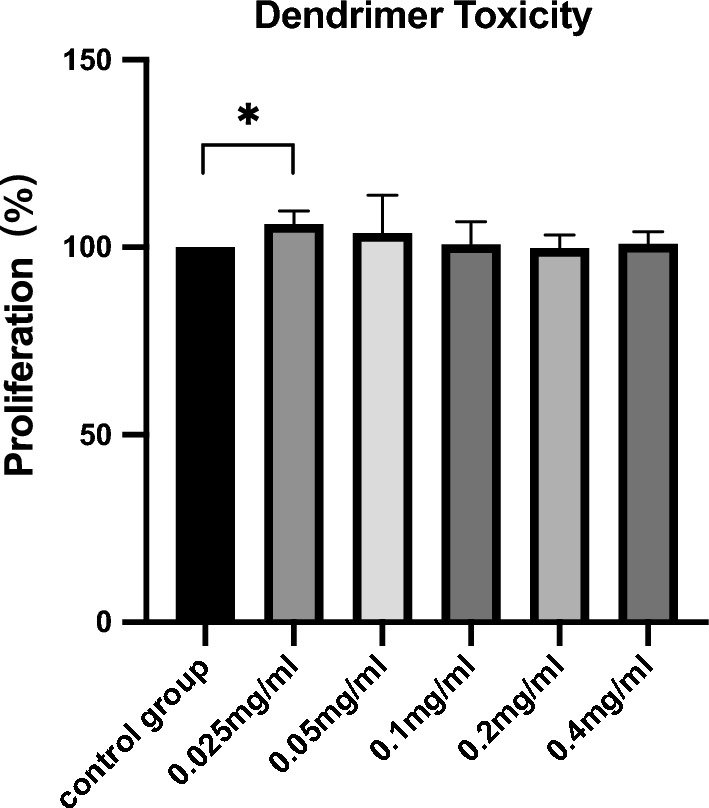
The toxicity of the dendrimer on SZ95 sebaceous gland cells was assessed by CCK-8 assay. Data are shown as mean ± standard deviation (SD), n = 3. *13cRA-D* dendrimer-conjugated isotretinoin

As assessed using the CCK-8 assay, the dendrimer mildly promoted the proliferation of sebaceous gland cells at concentrations ranging from 0.025 to 0.4 mg/mL, with the most notable effect observed at 0.025 mg/mL (p = 0.0400, Fig. 3). The dendrimer induced no toxicity toward sebaceous gland cells.

### *Sustained release of 13cRA-D *in vitro

**Fig. 4:**
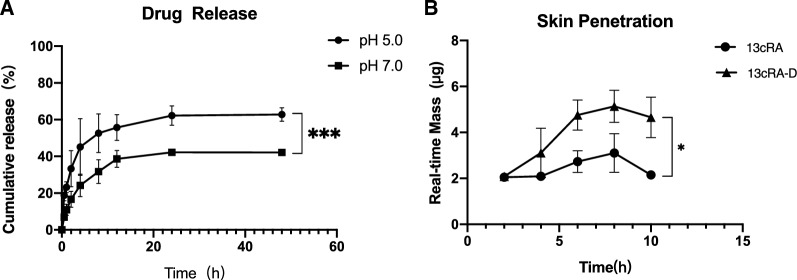
Controlled release properties of 13cRA-D. **A** Controlled release of isotretinoin from 13cRA-D at pH 5.0 and pH 7.0 in a hydroalcoholic solution over 48 h. **B** Penetration of 13cRA and 13cRA-D through the pig dermis, monitored over 10 h. Data are presented as the mean ± standard deviation (SD), n = 3. *13cRA-D* dendrimer-conjugated isotretinoin

Given that acids can affect ester hydrolysis, we performed drug release studies at two physiologically relevant pH levels. We found that 13cRA-D exhibited excellent controlled release properties (Fig. 4A), with initial release observed after 0.5 h at both pH 5.0 and 7.0. However, isotretinoin was rapidly released from 13cRA-D at pH 5.0. At pH 7.0, rapid drug release occurred after 4 h. Under both settings, complete drug release was observed at 24 h.

### Efficient transdermal delivery of 13cRA-D

As shown in Fig. 4B, isotretinoin demonstrated skin permeation after 8 h, as detected by HPLC. Our findings suggest that 13cRA-D can cross the epidermis more effectively than free isotretinoin, thereby delivering an increased amount of isotretinoin to the target region.

### Delivery by 13cRA-D enhanced isotretinoin skin retention

**Fig. 5:**
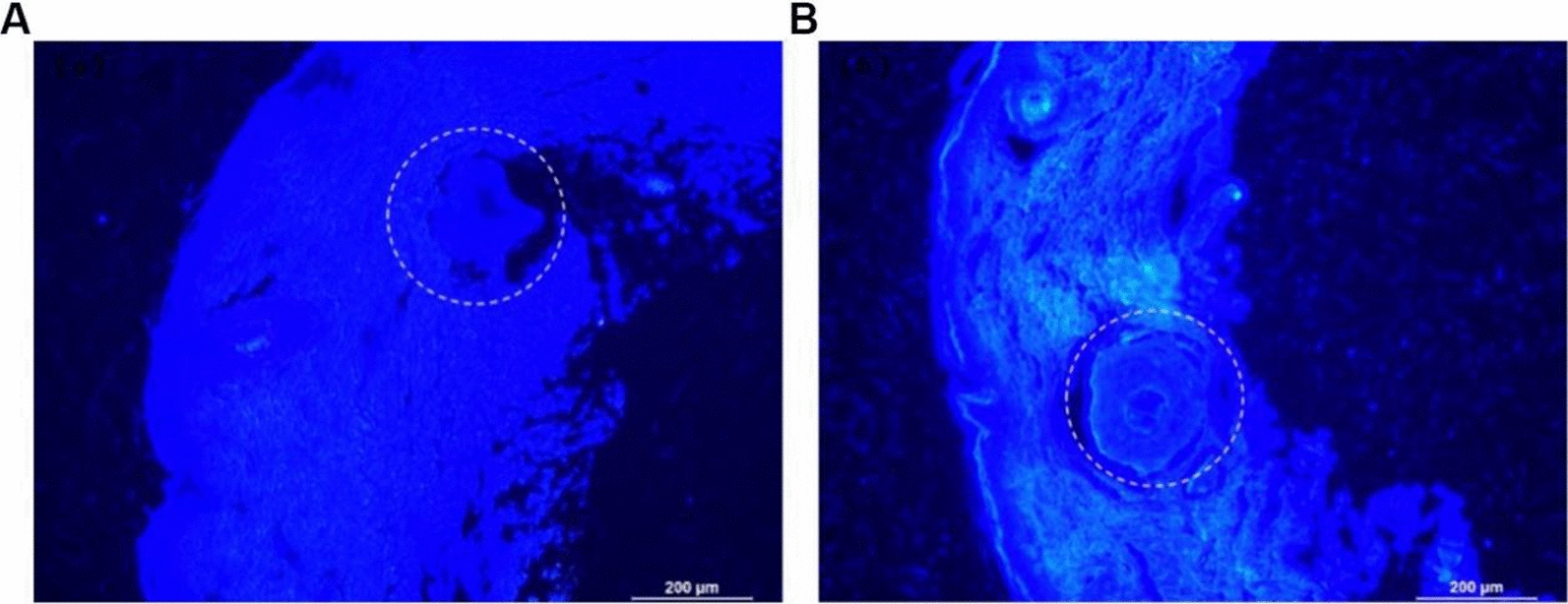
In vivo distribution of topically applied 13cRA-D. Distribution of 13cRA(**A**) and 13cRA-D (**B**) after incubation in a Franz diffusion cell for 10 h at 37 ℃ and 100 rpm. The dashed circle indicates the deposition of 13cRA. *13cRA-D* dendrimer-conjugated isotretinoin

Isotretinoin accumulation in the skin is crucial to treat skin diseases. Based on fluorescence microscopy assessments (Fig. 5A, B), 13cRA-D showed higher skin accumulation than free isotretinoin, with a more uniform distribution.

### 13cRA-D minimized skin irritation

**Fig. 6:**
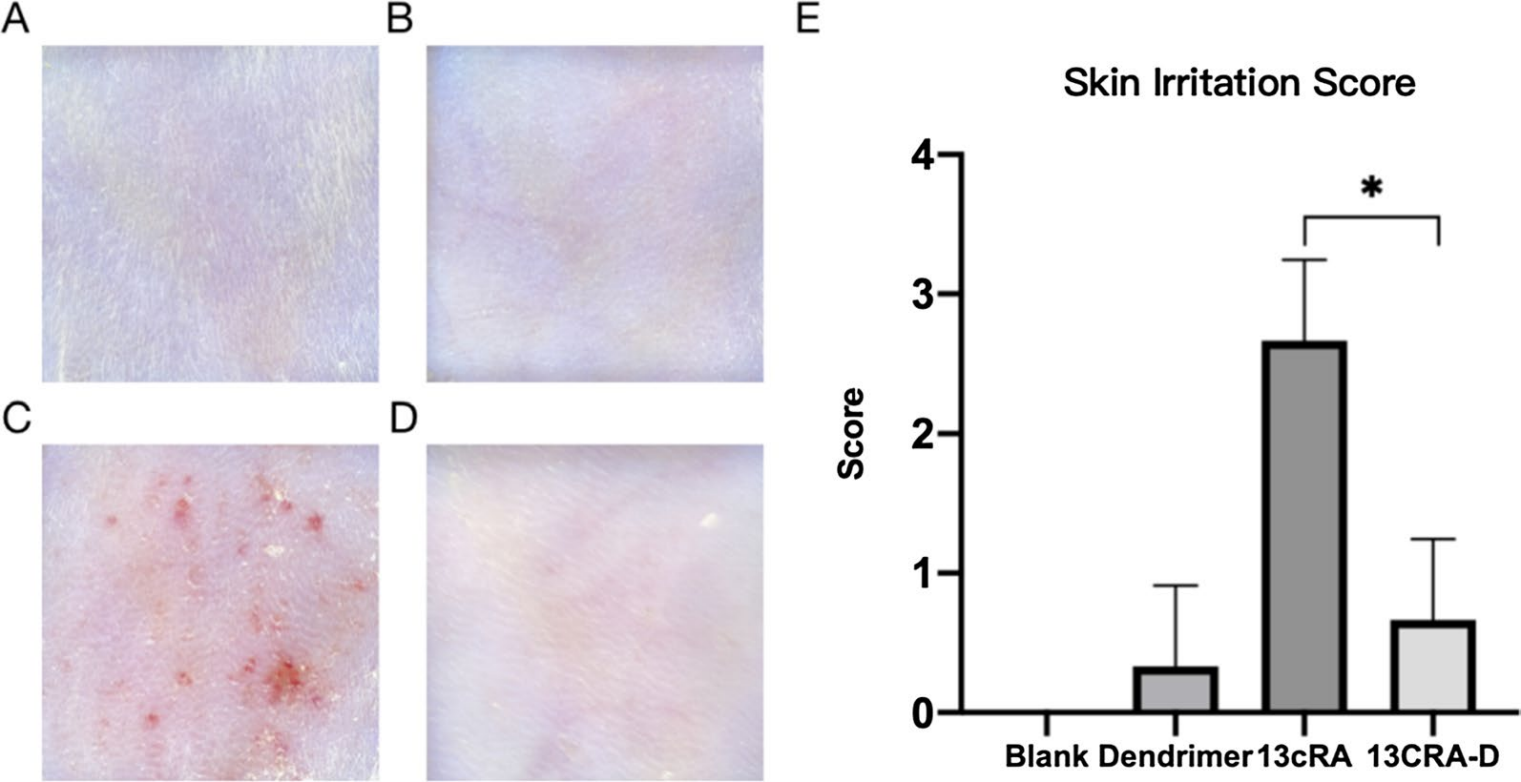
13cRA-D induces less skin irritation than free 13cRA. Representative images of the skin after application of phosphate-buffered saline (PBS) (**A**), Dendrimer(**B**),13cRA (**C**), or 13cRA-D (**D**) for 6 h at an approximate dose of 0.5 g. **E** Skin irritation scores. Data are presented as mean ± standard deviation (SD) (n = 3). *13cRA-D* dendrimer-conjugated isotretinoin

To evaluate the degree of irritation caused by PBS, dendrimers, 13cRA and 13cRA-D, we compared the severity of skin erythema after the application of respective compounds. Six hours post-application, we observed that 13cRA-D induced less skin irritation than free isotretinoin (Fig. 6). No erythema or slight erythema was observed in the 13cRA-D group, whereas treatment with free isotretinoin resulted in moderate-to-severe erythema (p = 0.013). No erythema was observed upon dendrimer application.

### 13cRA-D was more effective than free isotretinoin in an acne model

**Fig. 7:**
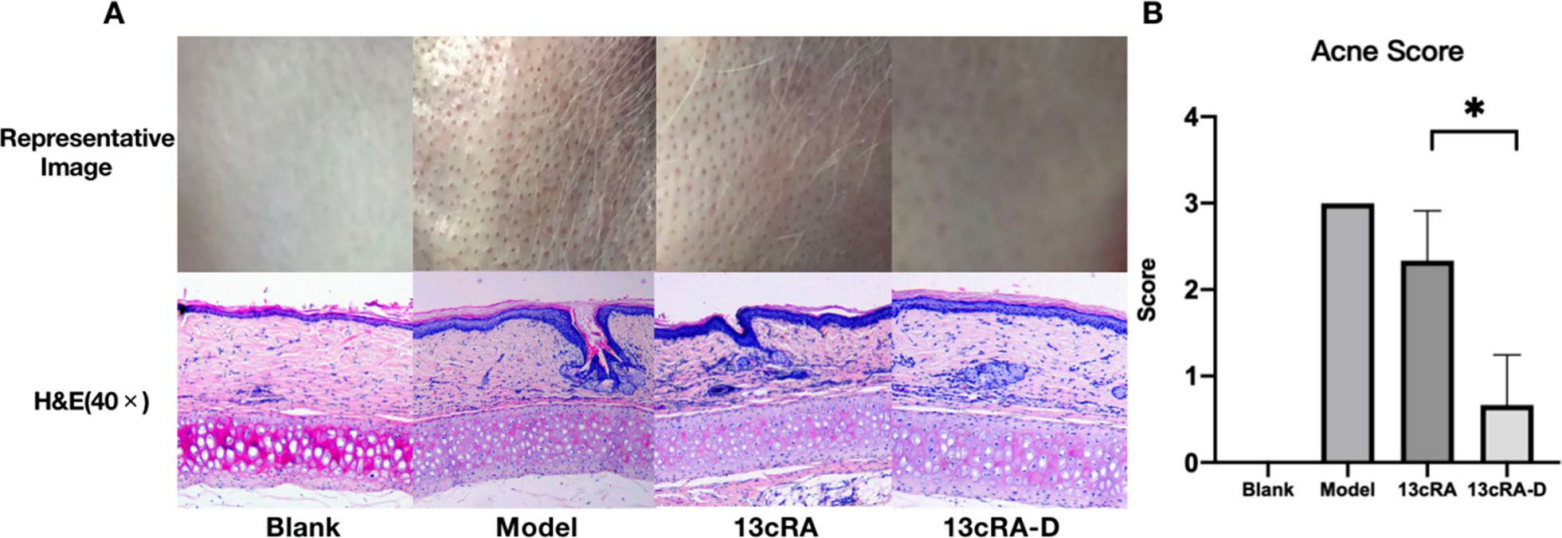
13cRA-D is more effective than free isotretinoin in an acne model. **A** Representative images and hematoxylin and eosin staining of the hyperkeratinization model. **B** Acne scores. Data are presented as mean ± standard deviation (SD), n = 3. *13cRA-D* dendrimer-conjugated isotretinoin

After 14 days of treatment, the 13cRA-D group showed almost no comedones, whereas 13cRA and untreated groups showed slight to moderate comedones (Fig. 7A). Based on the acne scoring (Fig. 7B), the 13cRA-D group displayed significantly less acne than the 13cRA group (p = 0.024). In addition, H&E staining revealed that 13cRA-D recipients had no follicular keratotic plugging. Hence, 13cRA-D performs better than free isotretinoin in the acne model.

### 13cRA-D demonstrated greater treatment efficiency than isotretinoin in a psoriasis model

**Fig. 8:**
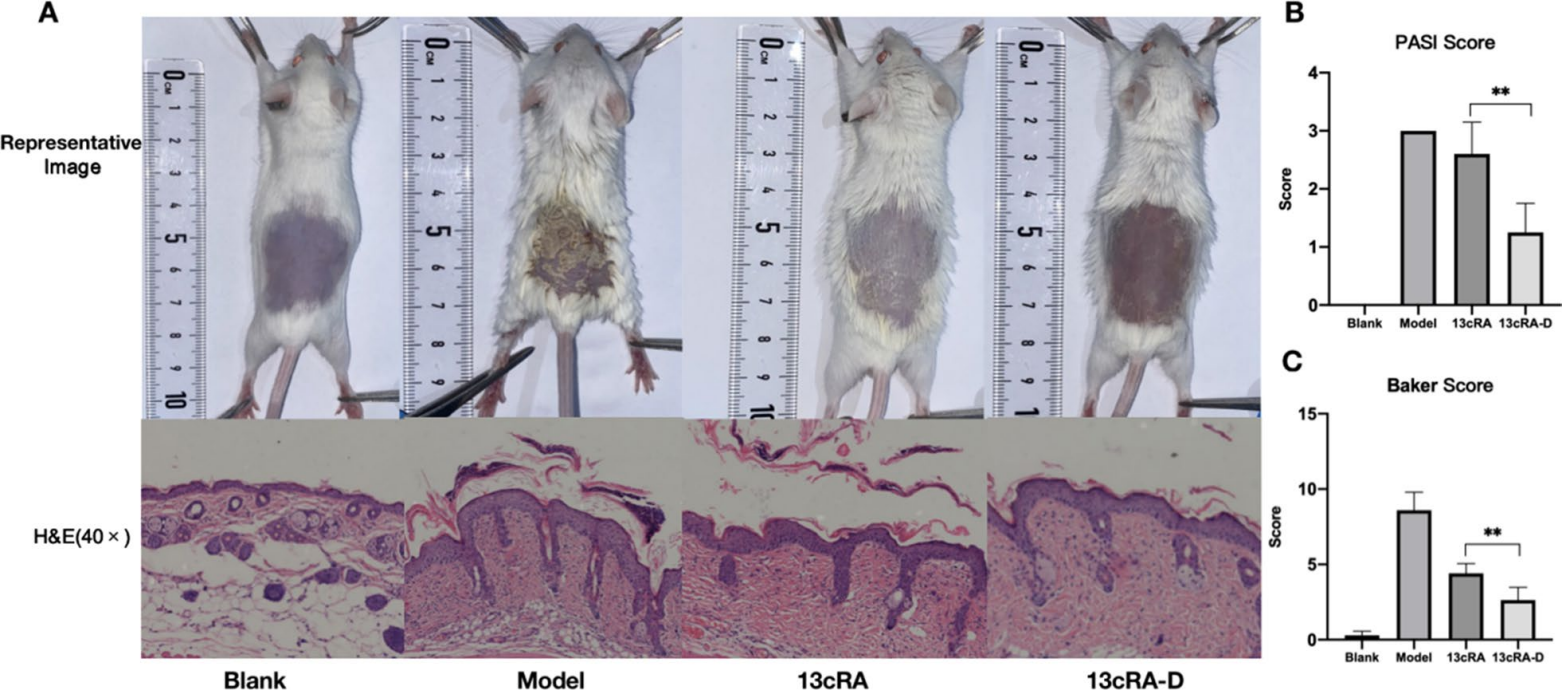
13cRA-D relieves psoriasis more effectively than 13cRA. **A** Representative photographs and H&E staining from the psoriasis model. Disease severity was assessed using PASI (**B**) and Baker score (**C**). *13cRA-D* dendrimer-conjugated isotretinoin, *H&E* hematoxylin and eosin, *PASI* Psoriasis Area Severity Index

As shown in Fig. 8A, 13cRA-D exerted a better therapeutic effect than free isotretinoin in the psoriasis model. On day 7, the control group displayed smooth skin with no scaling. The 13cRA and model groups exhibited notable scales and inflammatory infiltration. The 13cRA-D group showed a significant reduction in the number of scales. Compared with free isotretinoin, 13cRA-D significantly improved the skin condition, as determined by the PASI (p = 0.007; Fig. 8B) and Baker scores (p = 0.009; Fig. 8C).

## Discussion

Dendrimers can reportedly increase transdermal permeation and specific targeting of drugs [34]. The tetra-branched hydrophilic dendrimer was synthesized in a stepwise fashion, starting from a linear ethylene glycol chain to form a di-branched chain, followed by a subsequent reaction to obtain a tetra-branched hydrophilic dendrimer (1). By grafting (1) into hydrophobic 13cRA, 13cRA could form a water-soluble micellar structure coated with hydrophilic dendrimer chains owing to its amphiphilic characteristics [26]. We first established 13cRA-D, a new delivery system for isotretinoin, in which the drug is conjugated to a dendrimer via a hydrolytically degradable ester bond linkage. Notably, 13cRA-D rendered isotretinoin more stable and effective; hence, it could be an excellent delivery vehicle for isotretinoin.

Our skin permeation analysis demonstrated that 13cRA-D had better transdermal penetration than free isotretinoin, which poorly permeates the stratum corneum. 13cRA-D was present on the exterior surface of the dendrimer as a lipid-like hydrophilic structure, which is ideal for use as a drug delivery vehicle. The physical characteristics of 13cRA-D enabled it to reach the desired target more quickly and efficiently than free isotretinoin. In addition, 13cRA-D facilitated isotretinoin retention in the skin. The permeation and retention of a high drug concentration in the skin can be beneficial for treating several skin disorders. Importantly, uniform distribution may reduce side effects and enhance treatment efficacy.

In vivo, 13cRA-D effectively reduced skin irritation, indicating its potential as a topical formulation that minimizes isotretinoin-related side effects. In acne and psoriasis models, 13cRA-D significantly improved treatment efficacy. Accordingly, 13cRA-D may represent a new therapeutic option for multiple skin disorders, including acne and psoriasis.

**Fig. 9:**
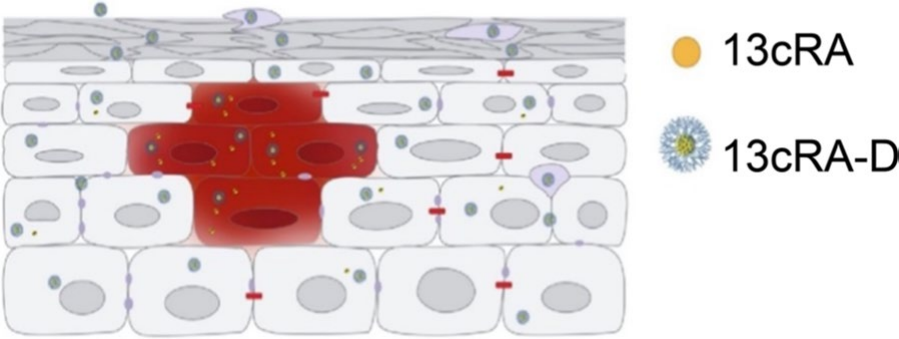
Proposed mechanism of 13cRA-D drug controlled release ability

The 13cRA-D formulation could control isotretinoin release, which is an ideal feature of a drug carrier. The dendrimer and isotretinoin are connected by a hydrolyzable ester bond, which is easier to break in an acidic environment than in a neutral one. Typically, the inflammatory environment is acidic owing to the accumulation of inflammatory factors [35]; therefore, 13cRA-D would be rapidly released in the inflammatory environment (Fig. 9). Because of this accumulation, the ester bond of 13cRA-D is more likely to be hydrolyzed at inflamed sites than in healthy tissues. This characteristic can increase the isotretinoin concentration, specifically at inflammatory sites, thereby delivering isotretinoin to the target area for precise and effective disease treatment. Given the multifunctional capacities, 13cRA-D may be a better choice for next-generation topical treatments for acne and psoriasis.

13cRA-D can efficiently permeate the skin and accelerate isotretinoin release at the site of inflammation.

In recent years, considerable efforts have been made to develop topical delivery systems for nano- and microparticles. Wang et al. loaded isotretinoin nanoparticles into human umbilical cord mesenchymal stem cells and showed that these nanoparticles efficiently controlled isotretinoin release and reduced side effects [36]. Future investigations need to compare the efficacy of different formulations for treating acne.

## Conclusion

Collectively, we demonstrated the benefits of coating isotretinoin with dendrimers for drug delivery and disease treatment. 13cRA-D controlled isotretinoin release, improved skin distribution, reduced side effects, and permeated the skin efficiently, positively impacting therapeutic efficacy. Accordingly, we demonstrated a new approach for isotretinoin application that could enhance the biological characteristics of isotretinoin and provide a more precise treatment of acne and psoriasis.

## Data Availability

Not applicable.

## Abbreviations

13cRA-D: Dendrimer-conjugated isotretinoin
PBS: Phosphate-buffered saline
SZ95: The human sebaceous gland cell line SZ95
MEM: Eagle’s Minimum Essential Medium
DMEM: Dulbecco’s Modified Eagle Medium
FBS: Fetal bovine serum
NMR: Nuclear magnetic resonance
TEM: Transmission electron microscopy
CCK-8: Cell Counting Kit-8
DCC: Dicyclohexylcarbodiimide
HPLC: High-performance liquid chromatography
H&E: Hematoxylin and eosin
PASI: Psoriasis Area Severity Index
